# The Environmental History of Cetaceans in Portugal: Ten Centuries of Whale and Dolphin Records

**DOI:** 10.1371/journal.pone.0023951

**Published:** 2011-09-09

**Authors:** Cristina Brito, Andreia Sousa

**Affiliations:** 1 Centre for Overseas History (CHAM), Faculdade de Ciências Sociais e Humanas, Universidade Nova Lisboa (FCSH-UNL), Lisboa, Portugal; 2 Escola de Mar, Edifício Instituto de Ciência Aplicade e Tecnologia (ICAT), Campus de Faculdade de Ciências da Universidade de Lisboa (FCUL), Lisboa, Portugal; National Institute of Water & Atmospheric Research, New Zealand

## Abstract

The history between cetaceans and humans is documented throughout time not only in reports, descriptions, and tales but also in legal documents, laws and regulations, and tithes. This wealth of information comes from the easy spotting and identification of individuals due to their large size, surface breathing, and conspicuous above water behaviour. This work is based on historical sources and accounts accounting for cetacean presence for the period between the 12th and 17th centuries, as well as scientific articles, newspapers, illustrations, maps, non-published scientific reports, and other grey literature from the 18th century onwards. Information on whale use in Portugal's mainland has been found since as early as the 12th century and has continued to be created throughout time. No certainty can be given for medieval and earlier events, but both scavenging of stranded whales or use of captured ones may have happened. There is an increasing number of accounts of sighted, stranded, used, or captured cetaceans throughout centuries which is clearly associated with a growing effort towards the study of these animals. Scientific Latin species denominations only started to be registered from the 18th century onwards, as a consequence of the evolution of natural sciences in Portugal and increasing interest from zoologists. After the 19th century, a larger number of observations were recorded, and from the 20th century to the present day, regular scientific records have been collected. Research on the environmental history of cetaceans in Portugal shows a several-centuries-old exploitation of whales and dolphins, as resources mainly for human consumption, followed in later centuries by descriptions of natural history documenting strandings and at sea encounters. Most cetaceans species currently thought to be present in Portuguese mainland waters were at some point historically recorded.

## Introduction

Environmental history is the history of the mutual interaction between humans and the rest of of the natural world [1]. It seeks understanding of human beings as they have lived, worked and thought in relation to nature through the changes brought by time [2]. Marine environmental history deals with this in relation to the sea, i.e., marine ecosystems and animals [3], [4] and is still at its infancy when compared to its terrestrial counterparts [5]. Commonly, the subjects studied are the evaluation of the impacts of changes caused by human agents in the natural environment and, reciprocally, the effect of the natural environment and populations on human societies and their histories [2]. It offers a multidisciplinary, or holistic, perspective on the long term interaction of human and marine life [6].

Basic sustenance is the origin of human exploitation of natural resources, historically including hunting, gathering, fishing, herding, and agriculture [2]. Hunters, herdsmen, farmers and fishers have always had a stock of practical knowledge about the world around them and often paid particular attention to those animals which did not seem to fit into the known categories [7]. Economy, trade, and world politics are regulated – whether humans wish it or not and whether or not they are conscious of it – by the availability, location, and finite nature of what, in language derived from economics, are called natural resources [2]. Whaling is part of this human survival history. Because whales are natural resources, their trade and its economy are also an important part of marine environmental history. But aside from these practical aspects, large whales and dolphins have always fascinated people, with myths and references to these marine animals dating from centuries back present all around the world in a multitude of human cultures [8]–[10].

Marine environmental history of cetaceans in Portugal remains to be written, with important records still to be reviewed. Our study is timely as no such review has been published for Portuguese waters and thus it fills an important historical gap. The main objective of this study was to review the magnitude of cetaceans' occurrence over the centuries in Portugal mainland considering a socio-cultural context and as a result to understand shifts in their presence in relation to human perceptions. We intended to broader contextualize their relevance to economic activities, as well as to the history of Portuguese natural history, marine scientific research and popular culture both in the past and nowadays.

Cetaceans are an ideal subject for the study of marine environmental history as well as in related fields of science and culture. To begin with, cetaceans are very well adapted to their aquatic habitats and play a significant role on their ecosystems, thus potentially acting as sentinels for the condition of their prey. Therefore, they provide ecological and environmental information on other links of their food chain and indications about the ecological conditions of the region they are living on. Cetaceans are also easily located and identified in the historiography, are good historical indicators and an useful subject for human and social sciences projects. Cetaceans are big animals, they come to the surface to breathe and show attractive and conspicuous surface behaviours such as speed movement in large groups, breaching, leaps, and breaths visible at long distances. All these characteristics result in a continued and global human fascination expressed in their inclusion in old maritime reports, early natural history descriptions, sea-related stories, as well as legal documents and laws. Portugal is no exception and all, from kings to fishermen, from navigators to naturalists, from biologists and researchers to students and the public at large, have shown an interest in cetaceans, thus creating a very particular historical relationship between us humans and them.

## Methods

### Historical and methodological framework

**Figure 1:**
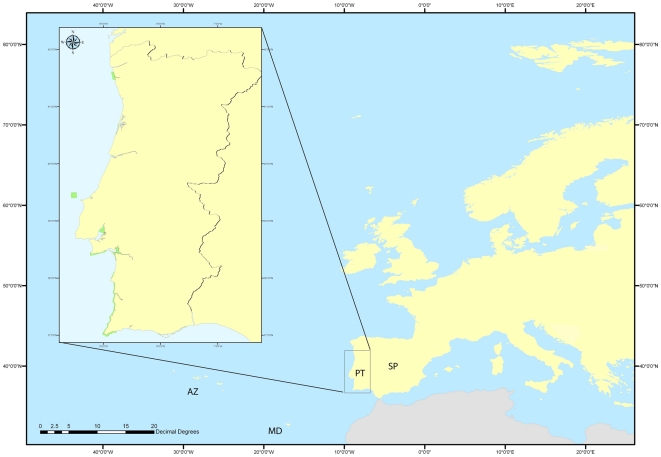
Map of Portugal, showing the mainland (PT) and the archipelagos of Madeira (MD) and the Azores (AZ) in the Atlantic Ocean. Marine protected areas are shown in green.

Portugal has its continental borders defined since 1143 [11], [12] (Figure 1). A search for sources that mentioned cetaceans' historical diversity and occurrence over the centuries and in different socio-cultural contexts on Portugal mainland was conducted. This research was based on the National Library of Portugal (Lisbon) records, but also on regional libraries and local historical archives (Peniche, Ericeira, Sesimbra, Faro). The search was designed to find and compile historical references to the occurrence of whales and dolphins during different types of activities or events, such as whaling (including indirect captures or by-catches), strandings, and sightings at sea. It included historical sources and accounts from the period between the 12^th^ and 17^th^ centuries and scientific articles, newspapers, illustrations, maps, non-published scientific reports and some other grey literature such as unpublished thesis from the 18^th^ century onwards. A first approach to Portuguese national laws that regulate fishing, maritime exploitations and several other human activities related to the presence of whales and dolphins was also conducted.

To establish a temporal frame, five time periods were considered reflecting both historical moments and cetacean related events in Portugal: a) a medieval period from the beginning of the 12th century, time of the first cetacean historical accounts, to the end of the 14th century when Portuguese overseas explorations peaked; b) a renaissance period, from early renaissance in the beginning of the 15th century to a late renaissance and enlightenment period in early 18th century; c) an 18th and 19th centuries period, from the year 1793, time of the first reported stranded large whale, to the year 1881 time of the first fishing statistic counting whale captures; d) a recent period containing most of the 20th century, from 1882 to 1975 which marks the beginning of continuous collection of cetaceans records from stranding events; and e) a modern period, from 1976 to 2010, reflecting the changes at the end of the 20th century and beginning of the 21st century.

Cetaceans' records in the historiography, i.e., a measure of the number of historical accounts in each period for each species, may be considered a way to measure their past presence in a region given that the number of historical sources reflects the importance of a certain historical event [13]. Accounts were classified under three types of activities or events - whaling, strandings and sightings – which reflect distinct interests of different groups of people towards these animals. The number of related scientific disciplines in each period was counted, as the development of environmental sciences offers a clear evidence of what we regard as knowledge of the world [7]. The number of historical accounts or scientific publications produced by Portuguese zoologists and researchers were also considered. A qualitative assessment of effort was used because it is necessary to relate historical accounts with the intensity of empirical, entrepreneur or scientific dedication to reporting the presence of whales and dolphins in the Portuguese shores. Uncertainties affect both the number of identified species and effort information [14]. Trying to account for a qualitative effort, number and type of entity as well as number of zoologists/scientist involved in obtaining information about cetaceans were listed and counted and a numeric value for each period was obtained to describe effort. A resume of all information collected is presented in Table 1.

**Table 1:** Description of all information related to species accounts in each time period.

Time frame	Geographical localization	Species accounted (N)	Type of account	Related scientific discipline (N)	Historical events	Effort for detectionn = researchers(N = total)
**12^th^–14^th^ century**	Central west and south coast	Whales; the black whale; dolphins (3)	Whale use; medieval whaling	Anatomy (1)	Direct and possibly intense captures and scavenging according to individual availability	Local fishing communities; the Portuguese kingdom (2)
**15^th^-Early 18^th^ century**	All along the coast	Whales and dolphins, still not specific identifications (2)	Whale use; Basque type whaling	Anatomy (1)	Decrease on number of available animals to capture; extinction of right whales.	Local fishing communities; the Portuguese kingdom (2)
**1723–1881**	Mainly central and south coast	Baleen whales; sperm whales; orcas; common dolphins; porpoises (4)	Dolphin captures; strandings and one mass stranding	Anatomy; Taxonomy (2)	First “naturalist” accounts of strandings; newspapers and illustrations	Local fishing communities; Interested people; naturalists and zoologists; the Portuguese kingdom (4)
**1882–1975**	All along the coast, still with special incidence on central west coast	*Phocoena phocoena; Delphinus delphis; Tursiops truncatus; Stenella coeruleoalba; Globicephala sp; Orcinus orca; Ziphius cavirostris; Physeter macrocephalus; Balaenoptera physalus; Balaenoptera acutorostrata; Balaenoptera borealis; Balaenopetra musculus; Megaptera novaeangliae *(13)	Early modern whaling; Industrial whaling; dolphin captures; strandings; natural observations	Anatomy; Taxonomy; Ecology (3)	Increase in the number of strandings and observations; increase of industrial captures and subsequent decrease of natural populations; great increase on species identification; first identification of a resident population of bottlenose dolphins	Local fishing communities; naturalists and zoologists 9; whalers; the public; the Portuguese government (13)
**1976–1998**	All along the coast	All the above, plus *Eubalaena glacialis*; *Grampus griseus*; *Pseudorca crassidens*; *Kogia breviceps*; *Mesoplodon *sp. (18)	Dolphin captures; strandings; natural observations; scientific research	Anatomy; Taxonomy; Ecology; Behavior; Acoustics; Conservation (6)	Increase in the number of strandings and observations; first scientific campaigns and conservation efforts; beginning of continuous study about the resident population; increase on biology and ecology knowledge no significant increase on species identification	Fishermen; biologists and researchers 14; the public; the Portuguese government (17)
**1999–2010**	All along the coast, particularly central west coast	All the above, except *Eubalaena glacialis* (17)	Scientific research and whale/dolphin watching	Anatomy; Taxonomy; Ecology; Behavior; Acoustics; Genetics; Human interactions; Conservation (8)	Great increase on the number of sightings but no increase on species identification; small increase on new knowledge	Biologists and researchers 34; whale watchers; the public; the Portuguese government (37)

Note: (N) in each table cell indicates the total number of references for species, scientific disciplines and effort for detection; n in each table cell indicates de number of researchers accountable for total effort.

## Results

### Historical occurrence of cetaceans

**Figure 2:**
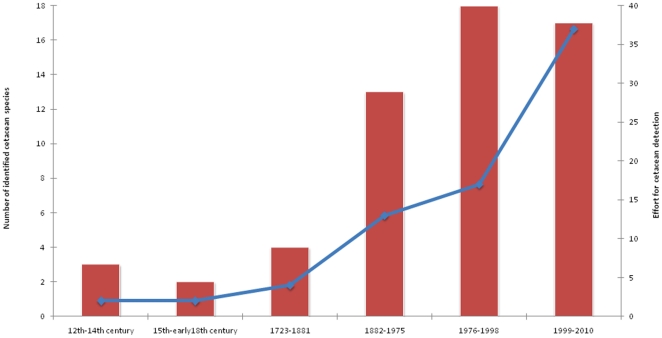
Number of identified cetacean species over time (bars), reflecting the knowledge gathered over time, across to effort for its detection (line).

Results show that, over the centuries, the number of identified (used, captured, stranded or observed) cetaceans' species greatly increased, accompanying the effort for its detection and the growing number of zoologists and of the public, and not only fishermen, dedicated to observe these animals and leading to accumulation of scientific and popular knowledge over time (Figure 2; see also Table 1).

The first account of a cetacean occurrence in Portugal dates back to the 12^th^ century and is the presence of the word “whaling” in several monastery books [15], [16], and references to taxes applied to captured “black whales” [15], most probably right whales (*Eubalaena glacialis*). This is also the first (and only for several centuries) written reference to a whale species. The earlier references to whale use in central and south coasts of Portugal date from the 12^th^ century, coeval to Basque whaling activities in the bay of Biscay [17], and are continuous ever since. No certainty can be given about medieval and early records, however preliminary evidence suggests that both scavenging of stranded whales or use of captured ones may have happened [17]. The only historical whale extinction for Portugal was the right whale; populations of this species started to be exploited by land based whaling since the 11^th^ century by Basque whalers as well as by Portuguese fishermen [17] and extinction of the population during the 17^th^ century was confirmed in the 1980 s by researchers [13]. In Portugal, the only 20^th^ century sighting of these whales occurred in February 1995 off Cape St. Vicente (south west coast), but there is no direct evidence whether the two animals (mother and calf) were remnants of the original north eastern Atlantic population or vagrant individuals [18].

**Figure 3:**
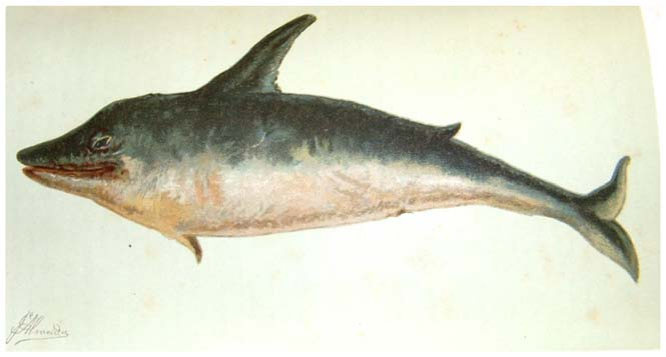
Drawing of a dolphin identified as *Delphinus maximus* occurring in the Portuguese shores from Silva (1891); the same illustration could represent a bottlenose dolphin (*Tursiops truncatus*), a common dolphin (*Delphinus delphis*) or even a harbor porpoise (*Phocoena phocoena*).

Common dolphins (*Delphinus delphis*) (Figure 3) and/or harbour porpoises (*Phocoena phocoena*) were also identified as soon as the 12^th^ century [19]. Considering that these are not large animals among cetaceans, the existence of early accounts likely means they were probably very common in the Portuguese coastline and most certainly were captured by fishermen. This is similar to what happened in Spain, where the human relationship with small cetaceans was characterized by its intensity, both due to continuous capture over the centuries (for consumption or due to its competition with fisheries) and, contrastingly, to protection of these cetaceans as they were indicators of nearshore fishing banks [20]. Both competition and cooperation between humans and dolphins are well established in their European history.

**Figure 4:**
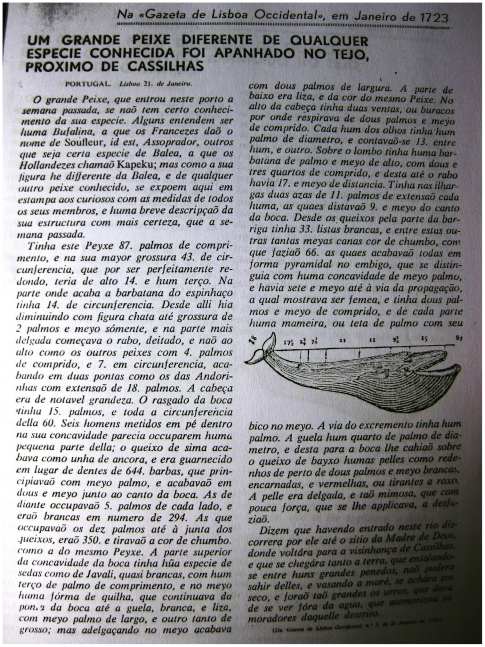
Portuguese newspaper “*Gazeta de Lisboa Occidental*”, dated 21st January 1723, presenting the stranding of a large whale with a very detailed description and an illustration of the animal.

The first identifiable large whale in Portuguese records corresponded to the stranding of a whale (later identified through its written description as a fin whale *Balaenoptera physalus*) in 1723 (Figure 4), followed by a sperm whale stranding in 1782 and the mass stranding of sperm whales (*Physeter macrocephalus*) in 1784 [21]. These are big and conspicuous animals, commonly observed only a few miles from the shore but whose strandings are easily spotted and reported. From that time forward records of strandings were typically accompanied by detailed description of anatomical features of the specimens. These whales were also one of the main targets of both early and recent industrial whaling and were recorded with special detail during the 20^th^ century whaling period.

**Figure 5:**
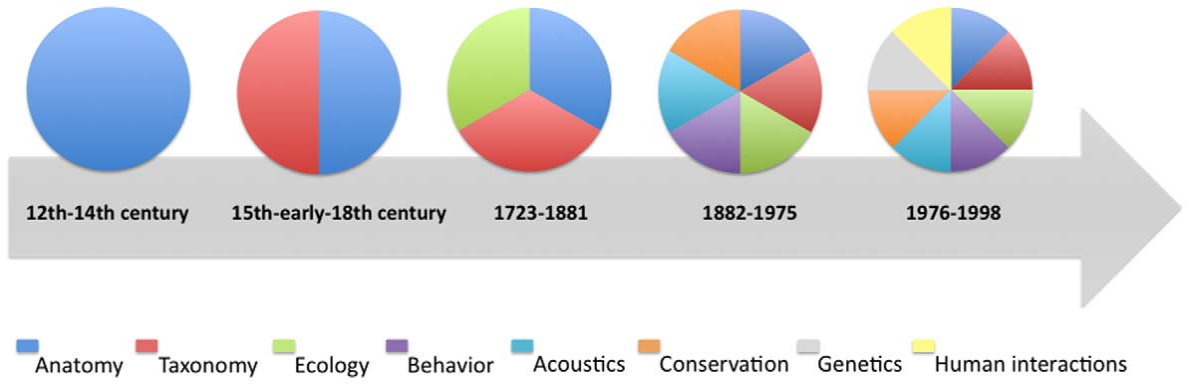
Number of different disciplines related to cetaceans throughout time (n = 8).

**Figure 6:**
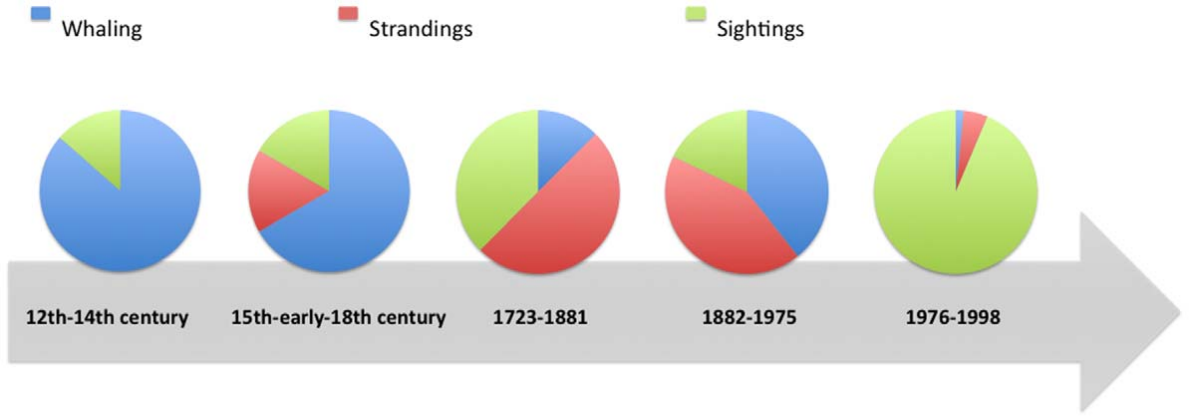
Number of historical and recent accounts for cetaceans over time (n = 142), including both historical sources and scientific publications, considering the three main activities related to cetacean presence (whaling, strandings and sightings).

After the early historical accounts, an important increase on cetacean knowledge in Portugal occurred in the late 19^th^ century and continued in the 20^th^ century, translated in the increase of number of species identified (due to zoologists observations but mainly to records from the 20^th^ century whaling period) as well as the increase of related disciplines and recent historical accounts (Figure 5). If in the first descriptions only anatomical aspects were briefly referred, by this time references to their distribution, ecology and behaviour were sometimes also included. The number of new reports on known cetaceans species stagnated in the last decade, whereas the number of disciplines on scientific areas involving the study of cetacean and the number of records showed a continuous increase until the present (Figure 6). The increase in number of disciplines and scientific publications on the subject is also an expression of the shift of human interest from whaling to scientific study during the late 20^th^ century. A recent review on the 20^th^ century presence of cetaceans [22] showed 1313 occurrences of great whales captured off central coast of Portugal (1925–1927 and 1944–1951) and a total of 45 observations of cetaceans in non-directed captures between 1976 and 1978. Also accounted were 45 observations of dolphins and whales in sea-sightings as a result of observations of opportunity, from 2002 to 2008. In 2007 and 2008 a total of 63 boat-based visual surveys were conducted from three different geographic locations and as a result 45 independent sightings of cetaceans were recorded [22]. The most frequent small cetaceans off the Portuguese mainland coast are the common dolphin as reflected by the three distinct approaches used in this study. Regarding whales the most common species is the fin whale as shown by whaling records [22]. Overall, the cetacean community along the central coast of Portugal is similar to the one found all along the Iberia shore.

### Historical marine laws

Besides information on occurrence, abundance and distribution of cetaceans in a certain location, historical accounts also allow for a timeline of marine related laws to be determined which give us an idea on types of activities related to cetacean presence. In a first approach, that still needs further research, a total of 16 cetacean laws were passed from the 12^th^ to the 21^st^ century (Table 2). For centuries, predation and consumption, economical and commercial interests were the only preoccupations concerning these animals and, consequently, laws reflect this circumstance. Also noteworthy is that whale and dolphin captures were sufficiently well organized and developed to warrant the levying of tithes in the feudal system of 13^th^-century Portugal [17]. In fact, medieval and early modern laws regulated the capture and distribution of whale parts as well as the rights to sea spoils which also included whales [17]. Most of modern (20^th^ century) regulations also refer to captures and industrial whaling [23].

**Table 2:** Compilation of national laws, tithes or rules referring to cetaceans.

Law description	Year	Main Topic	Reference
***«que a baleia negra seja almotaçada per esta guisa»***	12th century	Referring to the taxes applied to the capture of black whales	[15]
***Informação: «e o dito rendeiro deve a haver todas as baleias cocas busaranhas roazes sereas e todos os peixes semelhantes a estes que os baleeiros matarem»***	1335/1336	Information: “and the said tenant must have all the whales, *cocas*, *busaranhas*, *roazes sereas* and all the fish similar to those which the whalers kill”	[15]
***Carta a 28 de Setembro Er. 1378 (in) em que «concedeu todas as baleações do reino a Afonso Domingues em que se obrigava o fornecimento de sal necessário em troca de determinadas rendas anuais»***	1340	Letter from the King : “[he] conceded all the whaling of the Kingdom to Afonso Domingues, who was obliged to supply the salt required in exchange for the fixed annual revenues”	[15]
***Condições especiais de baleação para o Brasil***	1765	Whaling special conditions in Brazil	[23]
***Contratos reais de baleação em Portugal continental***	1765	Whaling royal contracts in Portugal mainland	[23]
***Contratos reais de baleação em Portugal continental***	1768	Whaling royal contracts in Portugal mainland	[23]
***Contratos reais de baleação em Portugal continental***	1774	Whaling royal contracts in Portugal mainland	[23]
***Contratos reais de baleação em Portugal continental***	1786	Whaling royal contracts in Portugal mainland	[23]
***«Alvará, ordenando que é livre preparar e armar navios para a pesca da baleia e preparo do azeite no alto mar, em todas as costas do reino, até ás do Brazil e nas de Moçambique, e naturalisando os pescadores de qualquer nação, servindo dez annos em navios portuguezes de pescarias volantes; e ordenando igualmente a liberdade das pescarias sedentárias em qualquer das ilhas de Cabo Verde.»***	1798	Whaling contracts and regulations for all the Portuguese possession in the overseas, from Brazil to Africa (including Mozambique, and special rules for the Cape Verde whale fisheries)	[23]
***«Alvará, dando por extincto o contrato da baleia, e ordenando a liberdade da pesca d'este cetáceo.»***	1801	Extinction of the royal contracts for whaling and giving liberty to all for the fishing of whales in Portugal mainland	[23]
***«Lei, concedendo certos benefícios aos navios, utensílios, e indivíduos que se empregarem na pesca da baleia.»***	1862	Benefits to the ships and fishermen dedicated to the whales fisheries in the Azores.	[23]
***«Lei, prorrogando por mais dez annos a lei de 26 de Maio de 1862 sobre a pescaria da baleia nos Açores.»***	1877	Prorogation for the 10 years more of the previous law	[23])
***«Portaria, regulando a execução das leis de 26 de maio de 1862 e 10 de Abril de 1877 acerca da pesca nacional da baleia.»***	1886	New regulation concerning the two previous laws with special reference to the national whaling	[23]
***«Regulamento de Protecção dos Mamíferos Marinhos na Zona Costeira e Zona Económica Exclusiva Continental Portuguesa.»***	1981	Conservation and protection of marine mammals for the Portuguese mainland coast	[34]
***«Actividade de Observação de Cetáceos nas águas de Portugal continental.»***	2006	Whale and dolphin watching in Portugal mainland	[35]
***«Plano de Acção para a Salvaguarda e Monitorização da População Residente de Roazes do Estuário do Sado.»***	2009	Conservation and monitoring of a resident population of bottlenose dolphins	[36]

Note: Includes early to recent whaling regulation and recent cetacean conservation (n = 16).

Legal considerations have evolved alongside mentalities and these have gone through significant changes regarding cetaceans use and most importantly their conservation. In Portugal whaling laws were substituted by more conservation oriented rules during the late 20^th^ century following a worldwide trend for the conservation of large whales and dolphins which began in the 1970 s.

New legislation for the protection of these animals was published in Portugal mainland first in 1981 prohibiting the capture and any type of use of marine mammals and then in 2006, regulating cetaceans' observation which includes recreational, scientific and touristic activities. These are still applicable in Portugal nowadays and are rigorously enforced in the two national marine protected areas, which are important regions of cetacean occurrence both historically and in recent times [19].

## Discussion

This review aims to be a useful baseline reference for cetacean environmental history in Portugal. We were able to show that cetaceans played an important economic role from early on which has shifted to research and conservation domains in recent times. Indeed, long before the concepts of science and nature conservation existed, whales and dolphins were an integral part of both Portuguese culture and economy and were regarded as marine resources and their profitable capture was the main interest [17]. In accordance, oldest records, from the 12^th^ to the 17^th^ century, refer to the captured or stranded animals [17], [21] with explicit identification of their use and economic value, which is similar to records of the same period in other North [24] and South European countries [20], [25]. The scavenging of stranded cetaceans was an ancestral practice in the Iberian Peninsula [25] and other parts of Europe [24] and during the Middle Ages their meat was not included in the *“morticinum”* (impure animal food sources that were not to be sacrificed or hunted by humans) which clearly states its importance as food source [25]. In Europe, all through the Middle Ages and up until the beginning of the Renaissance, identified individuals were usually referred to as whales, dolphins or “big fish” [10], [24], with only brief anatomic descriptions of their characteristics. In accounts for Portugal mainland, unlike those from some other European sources [24], no naturalistic considerations were usually made during this period.

Regular species denomination in Latin only started to be referred in written material from the 18^th^ century onwards, accompanying the evolution of natural sciences in Europe and increasing number of Portuguese zoologists interested in this thematic [21]. After the 19^th^ century, Portugal, following the general European tendency, saw this particular interest result in a larger number of cetacean's observations made in their natural environment. After the middle 20^th^ century, researchers, institutions and the general public also became more aware of the existence and relevance of these animals in Portuguese coastal waters.

Despite the public's great interest on their lives, humans and cetaceans share a long conflicting history concerning the use of marine environment [8]. Whaling was a historically important maritime activity in Portugal, bringing large profits to the kingdom and originating several references as well as distinct laws, tithes and rules [17]. Following a decrease during the 18^th^ and 19^th^ centuries, these activities again gained economic importance during the 20^th^ century when two distinct moments of industrial whaling (first during the 1920 s and then during the late 1940 s and the beginning of the 1950 s) occurred [26], [27] contributing to the knowledge of cetaceans' biology and anatomy, and establishing some scientific foundations for future conservation.

Somewhat regular sightings [28], [29] and other observations, such as strandings [30], also began being recorded in the late 19^th^ and early 20^th^ centuries, and this early modern scientific information is similar to recent data of species occurrence. Reports made by the Portuguese whaling industry, local fishing communities as well as those made by naturalists and in science journals of the 19^th^ and early 20^th^ century were of considerable relevance to the present knowledge of cetaceans, all giving an important contribution to modern day cetacean studies [30]. Most of the species accounted during 20^th^ century industrial whaling have been referred either by scientific sightings [30] or observations of opportunity [27], [22] and are similar to what can be sighted nowadays. The exception is the lower number of large cetaceans sighted which can be due to their overexploitation in previous decades resulting in a fewer occurrences at present or to their mainly offshore occurrence which may not be suitably surveyed in the more recent coastal research projects.

In Portugal, the increase in number of identified species over time, as well as number of sightings, does not reflect an increase in cetacean abundance but rather reflects the effort dedicated to the observation of these animals. Over the centuries, the number of accounts for observed, stranded, used or captured cetaceans' species increased, clearly as a result of a greater endeavour towards the identification of the animals and the attention given to this matter by different audiences. It is also apparent that the nature of human activities related to cetaceans changed, the number of researchers, scientific disciplines and publications increased significantly and the public gained new perspectives about cetaceans' conservation.

It is only after 1976 that the study of marine mammals in Portuguese coasts gains a certain scientific continuity [e.g. 31] with a basic stranding data collection system being implemented [21]. Also scientific surveys gaining some importance in number and in accuracy and the implementation of long term research projects started [e.g. 32]. It was only in 1981 that the first law for the protection and conservation of marine mammals was passed and after that the only way to obtain information about cetaceans was through scientific surveys.

No trends on cetacean's abundance can be estimated because a quantitative estimate cannot be made from old records, but it can be established that the same group of cetacean species have been present off Portugal over the last hundred years.

Many people would have had the casual opportunity to observe whales, but fishermen and sailors, due to the nature of their activities, naturally would have had the greatest exposure to these creatures, more so than authors and early zoologists. Cetaceans sightings from the coast must have been regular during the Middle Ages and early Renaissance period, when cetacean populations ten times greater than the ones existing today dominated the seas [24]. Individual whales or entire pods of dolphins may follow along the coast or even swim in shallow waters when following shoals of fish [24]. Similarly, more whales may have stranded in an age of larger whale populations.

Our main goals were not about number of records or number of individuals, so no abundance estimates are given, but to account for the diversity of species over time and how it was registered and how it changed as well as to understand shifts in human activities related to the presence of cetaceans. Our results on the environmental history of cetaceans in Portugal show a several centuries old exploitation of whales and dolphins as resources mainly for human consumption. These are chronologically followed by strandings records and on sea encounter records and finally studies which were all included in natural history studies. Historical sources make it clear that several species of whales and dolphins were encountered in their natural environment at sea, in coastal shallows, and stranded along shore. With only a few exceptions (probably just the extinction of the right whales) cetaceans species currently thought to be present, even though in reduced numbers, in Portuguese mainland waters were historically recorded. Characterization of local or regional cetacean communities over time must use as much as possible pristine references. In a region where sea related human activities have a long historical presence, such as in Portugal, it is particularly important to avoid the shifting baseline syndrome [33]. We should use available historical sources, not simply anecdotes but rather relevant contributions, to obtain a clear perception of historical presence and species biodiversity comparable to present day information [33], [4]. All different historical forms of exposure to cetaceans contributed to the perception of what they are and recent changes towards the protection and improved scientific research indicate a strong step towards the continued conservation of their natural populations. From strange to stranded animals on beaches or individuals captured on open sea for human consumption, to natural populations requiring study and preservation, whales and dolphins have been a constant presence in the Portuguese history.

## References

[1] Mcneill JR (2003). Observations on the nature and culture of environmental history.. History and Theory.

[2] Hughes JD (2006). What is Environmental History?.

[3] Schwerdtner Máñes K, Ferse SCA (2010). The history of makassan trepan fishing and trade.. PLoS ONE.

[4] Fortibuoni T, Libralato S, Raicevich S, Giovanardi O, Solidoro C (2010). Coding early naturalists' accounts into long-term fish community changes in the Adriatic Sea (1800–2000).. PLoS ONE.

[5] Thomas K (1991). Man and the natural world: Changing attitudes in England 1500–1800.

[6] Starkey DJ, Smith TD, Barnard M (2011). Fisheries and marina animal populations: Learning from the long term.. PLoS ONE.

[7] Bowler PJ (1992). The Fontana History of the Environmental Sciences.

[8] Cohat Y, Collet A (2001). Whales Giants of the Seas and Oceans..

[9] Constantine R, Perrin, William F, Würsig, Bernd e Thewissen JGM (2009). Folklore and legends.. Encyclopedia of Marine Mammals.

[10] Cazeil N (1998). Monstres marins..

[11] Oliveira Marques AH (1985). História de Portugal. Volume I–III.

[12] Marques AP (1990). Portugal e o Descobrimento Atlântico: Síntese e Cronologia..

[13] Aguilar A (1986). A review of old Basque whaling and its effect on the right whales (Eubalaena glacialis) of the North Atlantic.. Reports of the International Whaling Commission.

[14] Poulsen RT (2007). An environmental history of North Sea ling and cod fisheries, 1840–1914. Fiskeri-og Sofartsmuseets Studieserie.

[15] Castro A (1966). A evolução económica de Portugal nos séculos XII a XV.

[16] Viterbo JSR (1983). Elucidário das palavras, termos e frases que em Portugal antigamente se usaram e que hoje regularmente se ignoram..

[17] Brito C (2011). Medieval and early modern whaling in Portugal.. Anthrozoos.

[18] Martin AR, Walker FJ (1997). Sighting of a right whale (Eubalaena glacialis) with calf off S.W. Portugal.. Marine Mammal Science.

[19] Brito C, Vieira N (2010). Using historical accounts to assess the occurrence and distribution of small cetaceans in a poorly known area.. Journal of the Marine Biological Association of the United Kingdom.

[20] Hansen FV (2009). Pescadores y delfines en el norte de España: Historia de su interacción desde la Edad Media hasta el siglo XX.. Itsas Memoria Revista de estudios Marítimos del País Vasco.

[21] Sousa A, Brito C (2011). Historical strandings of cetaceans on the Portuguese coast: anecdotes, people and naturalists.. Marine Biodiversity Records.

[22] Brito C, Vieira N, Sá E, Carvalho I (2009). Cetaceans' occurrence off the west central Portugal coast: a compilation of data from whaling, observations of opportunity and boat-based surveys.. Journal of Marine Animals and Their Ecology.

[23] Silva BAA (1891). Estado Actual das Pescas em Portugal..

[24] Szabo VE (2008). Monstrous fishes and the mead-dark sea: Whaling in the medieval North Atlantic. The Northern World, Volume 35.

[25] Hansen FV (2010). Los balleneros en Galicia (siglos XIII al XX)..

[26] Brito C (2008). assessment of catch statistics during the land-based whaling in Portugal.. Marine Biodiversity Records.

[27] Brito C, Vieira N, Sá E, Carvalho I (2009). Cetaceans' occurrence off the west central Portugal coast: a compilation of data from whaling, observations of opportunity and boat-based surveys.. Journal of Marine Animals and Their Ecology.

[28] Nobre A (1935). Fauna Marinha de Portugal: Vertebrados (Mamíferos, Repteis e Peixes).

[29] Teixeira AM, Duguy R (1981). Observations de delphinidés dans les eaux cotieres Portugaises..

[30] Sequeira M, Inácio A, Silva MA, Reiner F (1996). Arrojamentos de mamíferos marinhos na costa continental portuguesa entre 1989 e 1994..

[31] Sequeira M (1988). Mamíferos marinhos da costa portuguesa..

[32] dos Santos ME (1998). Golfinhos-Roazes do Sado: Estudos de Sons e Comportamento..

[33] Pauly D (1995). Anecdotes and the shifting baseline syndrome of fisheries.. Trends in Ecology and Evolution.

[34] Decreto-Lei n°263/1981 De 3 de Setembro..

[35] Decreto–Lei n°9/2006 De 6 de Janeiro..

[36] Despacho n°21997/2009 De 2 de Outubro..

